# Quality Evaluation of Polar and Active Components in Crude and Processed *Fructus Corni* by Quantitative Analysis of Multicomponents with Single Marker

**DOI:** 10.1155/2016/6496840

**Published:** 2016-06-29

**Authors:** Yuhong Jiang, Hui Chen, Liling Wang, Jing Zou, Xiao Zheng, Zhihui Liu

**Affiliations:** ^1^College of Pharmacy, Nanjing University of Chinese Medicine, Nanjing 210023, China; ^2^Department of Pharmacy, Nanjing University of Chinese Medicine Affiliated Hospital, Nanjing 210029, China

## Abstract

*Objective*. To develop a quantitative analysis of multicomponents by single-marker (QAMS) method for the simultaneous determination of polar active components in* Fructus Corni*.* Methods*. Loganin was selected as the internal reference, and the relative correction factors (RCFs) of gallic acid, 5-hydroxymethyl-2-furfural, morroniside, sweroside, cornin, 7*α*-O-methylmorroniside, 7*β*-O-methylmorroniside, 7*α*-O-ethylmorroniside, 7*β*-O-ethylmorroniside, and cornuside were established. The contents of multicomponents were then calculated based on their RCFs, respectively. Contents of the 11 components were also calculated by external standard method and compared with those of the QAMS method.* Results.* The contents of the 11 components in 21 crude and 10 processed* Fructus Corni* products were measured. No significant difference was found in the quantitative results of the QAMS and external standard methods.* Conclusion*. QAMS could serve as an accurate and convenient method in determining the polar and active components in* Fructus Corni* and its processed products.

## 1. Introduction

*Fructus Corni* is derived from the dried ripe sarcocarp of* Cornus officinalis* Sieb. et Zucc (Cornaceae). In the practice of traditional medicine,* Fructus Corni* has a role in nourishing the liver and kidney, which provides the pharmacological basis for its wide use in many herbal prescriptions [1]. In particular, wine-prepared* Fructus Corni*, the major processed product of crude* Fructus Corni*, is frequently used in traditional medicine. Modern researches have shown that* Fructus Corni* has many chemical constituents, such as volatile constituents, iridoids, organic acids, carbohydrates, and tannins [2]. Among them, iridoid glycosides are a family of active ingredients in* Fructus Corni*, which are reported to have many pharmacological activities including immune regulation, glycemic lowering, anticoagulation, antiarrhythmia, neuroprotection, and memory improving [3–5]. In addition, gallic acid and 5-hydroxymethyl-2-furfural, which belong to organic acids, are reported to contribute to the effect of* Fructus Corni* in improving liver and kidney functions and related antioxidative effects [6, 7].

In the 2015 edition of Chinese Pharmacopoeia, only morroniside and loganin are used as indicators for quality control of* Fructus Corni*. Since several other iridoid compounds and components like organic acids also contribute to the integrative pharmacological effects of* Fructus Corni* and its processed products, quantification of two components alone may not be adequate for a comprehensive evaluation of herbal quality. To date, however, quantitative methods for the active components in* Fructus Corni* are largely compromised by the focus on a limited number of target components [8–10]. Therefore, there is a clear need for the development of a quality control method that may have more comprehensive coverage of active components from* Fructus Corni*.

High-performance liquid chromatography (HPLC) method is frequently used for the quality control of herbal drugs for its wide accessibility and good analytical performance. Quantitative analysis of multicomponents by single marker (QAMS) is a working strategy in the simultaneous determination of multiple components by HPLC. A predominant advantage of this strategy is the independence of the availability of all the target components, since their concentration could be easily calculated based on the relative correction factors (RCFs) in relation to the internal compound [11]. In this paper, we aimed to develop and validate a QAMS method for the simultaneous determination of 11 high-polarity active components from* Fructus Corni*. The results showed that, by choosing loganin as the internal reference substance and establishing the relative correction factors (RCFs) of other 10 components, this method proved reliable, accurate, and simple for determining those active components in* Fructus Corni *and its processed products.

## 2. Materials and Methods

### 2.1. Chemicals and Reagents

5-Hydroxymethyl-2-furfural, morroniside, sweroside, cornin, and cornuside were purchased from Nanjing Ningqi Pharmaceutical Co. Ltd. (Nanjing, China). Gallic acid and loganin were purchased from National Institute for Food and Drug Control (Beijing, China). The purity for each reference compound was greater than 98% as validated by HPLC analysis. 7*α*-O-Methylmorroniside, 7*β*-O-methylmorroniside, 7*α*-O-ethylmorroniside, and 7*β*-O-ethylmorroniside were prepared by our lab and structurally characterized based on ^1^H-NMR and ^13^C-NMR spectra. Their contents were 99.7% and 99.6% as determined by HPLC analysis, respectively. Acetonitrile of HPLC grade was purchased from Merck (Darmstadt, Germany). Formic acid of analytical grade was purchased from Roe scientific Inc. (USA). A Milli-Q water (Millipore, Inc., USA) purification system was used to obtain purified water for the HPLC analysis.

### 2.2. Herbal Plant

Crude* Fructus Corni* (numbers 1–21) was purchased from Anhui Xiehecheng Pharmaceutical Preparation Co., Ltd. (Bozhou, China) (batch numbers 131219, 131222, 140212, 140218, and 140403 were collected from Hubei Province; 140301, 140305, 140401, 140403, and 140528 were from Zhejiang Province; 140102, 140406, and 130616 were from Henan Province; 131219, 140102, 140301, and 140406 were from Jiangxi Province; 130616, 140301, 140305, and 140406 were from Anhui Province). Processed* Fructus Corni* (numbers 22–31) were also purchased from Anhui Xiehecheng Pharmaceutical Preparation Co., Ltd. (Bozhou, China) (batch numbers 131201, 140201, and 140506 were from Anhui Province; 131211 and 140216 were from Hubei Province; 131220, 140103, 140303, and 140407 were from Jiangxi Province; 140404 was from Zhejiang Province).

### 2.3. Instruments and Chromatographic Conditions

Analysis was performed on three different HPLC systems: Agilent 1100 series, including vacuum degasser (G1322A), quaternary pump (G1311A), autosampler (G1316A), and a ChemStation Workstation with VWD detector; Agilent 1260 series, including DAD detector and Agilent ChemStation Workstation; Waters 2695-2996 high-performance liquid chromatography, including 2998PDA detector and Empower workstation. The chromatographic separation was performed on Elite C_18_ (4.6 mm × 250 mm, 5 *μ*m), Agilent C_18_ (4.6 mm × 250 mm, 5 *μ*m), and Welch Materials C_18_ (4.6 mm × 250 mm, 5 *μ*m).

**Figure 1:**
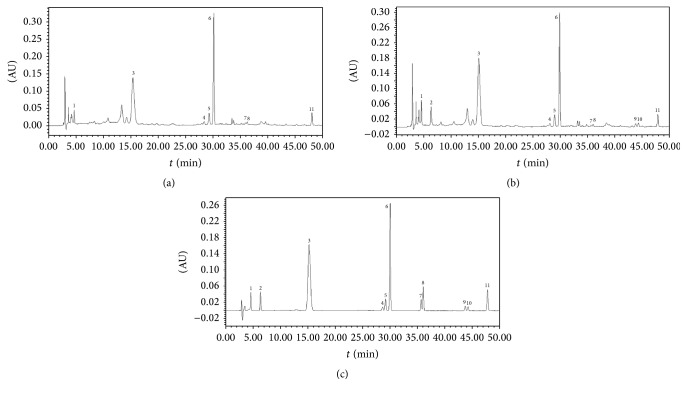
A chromatogram of crude* Fructus Corni* (a) and processed* Fructus Corni* (b); a chromatogram of mixture of standard compounds (c). 1: gallic acid, 2: 5-hydroxymethyl-2-furfural, 3: morroniside, 4: cornin, 5: sweroside, 6: loganin, 7: 7*α*-O-methylmorroniside, 8: 7*β*-O-methylmorroniside, 9: 7*α*-O-ethylmorroniside, 10: 7*β*-O-ethylmorroniside, and 11: cornuside.

The mobile phase was composed of 0.05% formic acid-water (A) and acetonitrile (B); the gradient program was shown in Table 1. Column temperature was maintained at 30°C. The flow rate was kept at 1 mL·min^−1^. The wavelength UV detection was set at 240 nm, and the injection volume was 10 *μ*L. Under the above chromatographic conditions, all the components were well separated (as shown in Figure 1).

### 2.4. Preparation of Standard Solutions

A mixed stock solution containing reference standards was prepared by dissolving weighed accurately samples of each compound in methanol, which obtained gallic acid 124.44 *μ*g·mL^−1^, 5-hydroxymethyl-2-furfural 120.12 *μ*g·mL^−1^, morroniside 720.3 *μ*g·mL^−1^, sweroside 60.66 *μ*g·mL^−1^, cornin 22.74 *μ*g·mL^−1^, loganin 415.2 *μ*g·mL^−1^, 7*α*-O-methylmorroniside 40.2 *μ*g·mL^−1^, 7*β*-O-methylmorroniside 81.52 *μ*g·mL^−1^, 7*α*-O-ethylmorroniside 20.16 *μ*g·mL^−1^, 7*β*-O-ethylmorroniside 20.12 *μ*g·mL^−1^, and cornuside 108.64 *μ*g·mL^−1^. The further dilution with methanol gives at least six different concentrations for the establishment of calibration curves. All stock and working solutions of standards were all prepared in dark brown bottles at 4°C until analysis.

### 2.5. Preparation of Sample Solution

The powder of* Fructus Corni* samples was precisely weighed (2 g) and immersed in 50 mL of methanol. Additional methanol was added to make up the loss after ultrasonic extraction for 30 min. All solutions were filtered through 0.45 *μ*m filter membranes before being injected into the HPLC system.

## 3. Results and Discussion

### 3.1. Method Validation

#### 3.1.1. Linearity

All the above six different concentrations of mixed standard solutions were injected (10 *μ*L) and calculated; each concentration was measured twice and took the average. The calibration curves were produced by plotting the integrated peak areas (*Y*) versus the concentration of each component (*X*, *μ*g·mL^−1^) and by the linear regression analysis. As was shown in Table 2, mixed standard solutions were in a certain range of linear quality.

#### 3.1.2. Precision

The same mixed standard solution was injected six consecutive times under chromatographic conditions and their RSD values were calculated. The RSD of gallic acid, 5-hydroxymethyl-2-furfural, morroniside, cornin, sweroside, loganin, 7*α*-O-methylmorroniside, 7*β*-O-methylmorroniside, 7*α*-O-ethylmorroniside, 7*β*-O-ethylmorroniside, and cornuside were 0.73%, 1.66%, 1.79%, 2.11%, 1.99%, 1.59%, 1.41%, 1.25%, 1.76%, 2.67%, and 3.31%, which indicated that the developed method had a well precision.

#### 3.1.3. Repeatability

Six samples (processed* Fructus Corni*, number 23) were prepared independently for checking the repeatability. The RSD of gallic acid, 5-hydroxymethyl-2-furfural, morroniside, cornin, sweroside, loganin, 7*α*-O-methylmorroniside, 7*β*-O-methylmorroniside, 7*α*-O-ethylmorroniside, 7*β*-O-ethylmorroniside, and cornuside were 4.05%, 2.60%, 1.60%, 0.97%, 0.93%, 1.43%, 0.68%, 0.69%, 1.10%, 1.52%, and 1.01%. The results showed that the method had good reproducibility.

#### 3.1.4. Stability

The stability of the same sample solution was analyzed at 0, 4, 8, 10, 12, and 24 h at room temperature. The RSD of gallic acid, 5-hydroxymethyl-2-furfural, morroniside, cornin, sweroside, loganin, 7*α*-O-methylmorroniside, 7*β*-O-methylmorroniside, 7*α*-O-ethylmorroniside, 7*β*-O-ethylmorroniside, and cornuside were 1.94%, 3.49%, 0.49%, 2.03%, 1.46%, 0.12%, 1.65%, 1.01%, 2.87%, 2.08%, and 2.30%, respectively. The results showed that the sample solutions were stable within 24 h.

#### 3.1.5. Recovery

Recovery test was carried out by adding reference substance solutions into a certain amount of processed* Fructus Corni* sample and analyzed. Six replicates were performed for the determination. The recoveries of the 11 compounds were shown in Table 3.

### 3.2. Calculation of Relative Correction Factors (RCFs)

Loganin was selected as the internal reference substance, and the RCFs are a constant of proportionality in a computational formula and can be calculated as follows: *f*_*is*_ = *f*_*i*_/*f*_*s*_ = (*C*_*i*_/*A*_*i*_)/(*C*_*s*_/*A*_*s*_) = (*C*_*i*_ × *A*_*s*_)/(*C*_*s*_ × *A*_*i*_) [12], where *i* is a compound, *s* is the internal reference substance, *C* is the concentration of compound, and *A* is the peak area. The RCFs of different components to loganin were calculated, respectively, and the results were shown in Table 4.

#### 3.2.1. Reproducibility of RCFs

*Influence of Different Instruments and Columns on RCF Values.* In this paper, we injected the mixed standard solutions diluted 2, 4, and 8 times and calculated RCFs of each component. The experiment was varied on three different instruments (Waters 2695-2998, Agilent 1100, and Agilent 1260) and three different columns (Elite C_18_, Agilent C_18_, and Wel Materials C_18_); results were shown in Table 5.

*Influence of Different Columns Temperature on RCF Values*. In this paper, analysis was performed on Waters 2695-2998 and Elite C_18_, with three different temperatures. Results were shown in Table 6. 

*Influence of Different Flow Rate on RCF Values*. In this paper, analysis was performed on Waters 2695-2998 and Elite C_18_, with three different flow rates. Results were shown in Table 7.

#### 3.2.2. Location of Target Chromatographic Peaks

The parameter of relative retention time was used to locate the target peaks. Using loganin as the external standard, the relative retentions between the other target marker and loganin were obtained in different columns and HPLC systems. Results were shown in Table 8.

#### 3.2.3. Comparison of the Quantitative Results between QAMS Method and External Standard Method

A total of 31 samples were injected for HPLC analysis. The contents of each component were calculated by QAMS method and external standard method; results were shown in Tables 9(a) and 9(b).

### 3.3. Discussion

*Fructus Corni* has been widely used in clinical practice for thousands of years and has well-known effects on kidney and good safety record [13]. At present, there were many reports about quantitative determination of the content of* Fructus Corni* and its processed products. However, most of those studies only focused on one or several few components. There were also some reports about the fingerprints of* Fructus Corni*, which were mainly used for qualitative analysis, but those methods could not be repeated [14–17]. In our laboratory, a method for simultaneous identification and determination of the 11 compounds in* Fructus Corni* using LC-MS/MS was established and validated, but LC-MS/MS method has not yet been fully popularized. In consideration of these facts, it was our striving direction to establish a quantitative analysis of multicomponents by single-marker method for the simultaneous determination of important and active components in* Fructus Corni *products, which was especially achievable by HPLC. In this study, we have successfully established QAMS method and realized the quantitative determination of major active components in* Fructus Corni *and its products.

In this paper, all the target components were iridoid glycoside components, except 5-hydroxymethylfurfural and morroniside. Loganin belongs to iridoid glycoside, which is also one of the index components in quality control of crude* Fructus Corni* and processed products in the 2015 edition of Chinese Pharmacopoeia. Loganin exhibits a high content in* Fructus Corni* and stable chemical property, in addition to easy accessibility. In comparison, several other iridoid glycoside components were relatively expensive, and some iridoid isomers (e.g., 7*α*-O-methylmorroniside, 7*β*-O-methylmorroniside, 7*α*-O-ethylmorroniside, and 7*β*-O-ethylmorroniside) were not readily available in the market. Taking these into account, loganin was selected as the internal standard in the development of the QAMS method.

When the QAMS method was applied for quantitative determination, only the internal content needs to be prepared with standard solutions, and the peaks of other components could not be directly confirmed. Therefore, how to locate the chromatographic locations of other target components accurately was one of the key steps for achieving the QAMS determination. In our study, we used the relative retention time between loganin and other components to locate their chromatographic peaks. Previous studies have proved that this method was simple and accurate, especially when it was used for those compounds featuring similar chemical properties [18]. As shown in Table 8, the relative retention times of all the compounds were below 5%, which indicated that this method can locate chromatographic peaks accurately.

Efficient separation of the numerous target components of* Fructus Corni* by HPLC proved challenging, and currently there were few reports for reference, which largely suffered from narrow coverage and poor repeatability. To overcome this difficult issue, in this study, several different mobile phase systems were therefore tried, including methanol-water, methanol-water containing phosphoric acid or formic acid, acetonitrile-water, and acetonitrile-water containing phosphoric acid or formic acid, with different gradient elution system [14–17, 19]. The results showed acetonitrile-water containing 0.05% formic acid system produced good separation and repeatability. In contrast, other mobile phase systems have many problems, such that the target components could not be separated at the same time, and detection time was too long. It could improve the peak shape and separation effect, prevent the peak tailing, and make the peak sharp and symmetrical. In this paper, this method shows good separation of eleven compounds in 50 mins by the HPLC system, which had greatly shortened the detection time and reflected the convenience and feasibility of the QAMS method. To inspect the influence of different flow rate on RCF values, we set up three different flow velocities: 0.9 mL·min^−1^, 1.0 mL·min^−1^, and 1.1 mL·min^−1^. The results were shown in Table 7; RSD values were all less than 5%. To inspect the influence of different columns temperature on RCFs values, we set the temperature to 25°C and 30°C, and each component could be separated; the RSD value was below 5%. But when the columns temperature was 35°C, only sweroside and cornin could not be separated. In addition, the experiment was varied on three different instruments (Waters 2695-2998, Agilent 1100, and Agilent 1260) and three different columns (Elite C_18_, Agilent C_18_, and Wel Materials C_18_) to investigate the influence of different instruments and columns on RCF values. As showed, in addition the Agilent C_18_ (4.6 mm × 250 mm, 5 *μ*m) column could not be used in Agilent 1260 (sweroside and cornin could not be separated completely); the other instruments and columns had good RSD values. Therefore, in order to ensure the accuracy of the RCFs of components by QAMS methods, we should prescribe the type of columns, HPLC's liquid phase system, or columns temperature, so that we could obtain satisfactory results.

## 4. Conclusion 

In this paper, we determined the content of components by external standard method at first; the QAMS method was used to calculate their contents, and we compared the two methods to verify the accuracy of QAMS method for quality control of* Fructus Corni* and its processed products. The results showed that there was no significant difference between the two methods. The QAMS method may provide a convenient, fast, and accurate approach in the quality control of traditional Chinese medicine.

## Figures and Tables

**Table 1:** Conditions of the mobile phase gradient.

*t*/min	A/%	B/%
0	93	7
6	93	7
8	92	8
20	92	8
30	85	15
50	78	22

**Table 2:** Standard curves of 11 components in *Fructus Corni*.

Compound	Regression equation	*r*	Linearity/*μ*g·mL^−1^
Gallic acid	*y* = 6411.2*x* − 13176	0.9999	3.89–124.44
5-Hydroxymethylfurfural	*y* = 7856*x* − 7229	0.9999	3.75–120.12
Morroniside	*y* = 14534*x* − 64815	0.9999	22.51–720.30
Cornin	*y* = 10795*x* − 3398.3	0.9995	0.71–22.74
Sweroside	*y* = 13446*x* − 7742.1	0.9998	1.90–60.66
Loganin	*y* = 14317*x* − 32209	0.9999	12.97–415.20
7*α*-O-Methylmorroniside	*y* = 12640*x* − 3224.1	0.9999	1.26–40.20
7*β*-O-Methylmorroniside	*y* = 15128*x* − 9720.8	0.9998	2.55–81.52
7*α*-O-Ethylmorroniside	*y* = 14273*x* − 283.77	0.9999	0.63–20.16
7*β*-O-Ethylmorroniside	*y* = 12527*x* − 1411.6	0.9998	0.63–20.12
Cornuside	*y* = 12461*x* − 11069	0.9999	3.39–108.64

**Table 3:** The results of recovery of eleven components in samples (*n* = 6).

Compound	Original/mg	Added amount/mg	Detected amount/mg	Recovery/%	RSD %
Gallic acid	0.030	0.031	0.064	108.15	1.18
5-Hydroxymethylfurfural	0.033	0.033	0.068	105.29	1.48
Morroniside	0.20	0.18	0.384	102.07	1.90
Cornin	0.0017	0.0019	0.004	101.21	4.88
Sweroside	0.016	0.017	0.034	108.73	1.55
Loganin	0.12	0.114	0.239	104.27	0.42
7*α*-O-Methylmorroniside	0.0059	0.0061	0.012	103.67	3.47
7*β*-O-Methylmorroniside	0.0126	0.013	0.025	97.25	1.59
7*α*-O-Ethylmorroniside	0.0049	0.0051	0.010	95.24	3.40
7*β*-O-Ethylmorroniside	0.0053	0.0057	0.011	105.66	1.60
Cornuside	0.025	0.024	0.049	98.54	0.64

**Table 4:** RCFs of each component in *Fructus Corni *(*n* = 6).

Mixed standard solutions	Gallic acid	5-Hydroxymethylfurfural	Morroniside	Cornin	Sweroside	7*α*-O-Methylmorroniside	7*β*-O-Methylmorroniside	7*α*-O-Ethylmorroniside	7*β*-O-Ethylmorroniside	Cornuside
1	2.253	1.824	0.985	1.327	1.067	1.131	0.947	1.003	1.146	1.151
2	2.311	1.841	0.990	1.386	1.083	1.146	0.956	0.978	1.128	1.159
3	2.333	1.841	0.989	1.428	1.098	1.139	0.977	0.974	1.164	1.170
4	2.415	1.835	0.992	1.386	1.076	1.138	0.961	0.968	1.158	1.184
5	2.443	1.853	0.986	1.377	1.140	1.115	0.948	0.953	1.238	1.189
6	2.159	2.036	1.004	1.334	1.150	1.145	0.957	1.015	1.006	1.185
Mean	2.319	1.872	0.991	1.373	1.102	1.136	0.957	0.982	1.150	1.173
RSD %	4.518	4.328	0.689	2.715	3.178	1.008	1.127	2.334	4.860	1.317

**Table 5:** RCFs by different instruments and columns (*n* = 3).

Instruments	Chromatograph columns	*f*_gallic acid_/*f*_loganin_	*f*_5-HMF_/*f*_loganin_	*f*_morroniside_/ *f*_loganin_	*f*_cornin_/ *f*_loganin_	*f*_sweroside_/ *f*_loganin_	*f*_7α-O-methylmorroniside_/ *f*_loganin_	*f*_7β-O-methylmorroniside_/ *f*_loganin_	*f*_7α-O-ethylmorroniside_/ *f*_loganin_	*f*_7*β*-O-ethylmorroniside_/ *f*_loganin_	*f*_cornuside_/ *f*_loganin_
Waters 2695-2998	Elite	2.44	1.87	0.99	1.37	1.10	1.14	0.96	0.98	1.15	1.17
	Agilent	2.61	1.83	1.03	1.50	1.19	1.21	0.94	0.92	1.10	1.10
	Wel Materials	2.68	1.85	0.99	1.44	1.18	1.18	0.87	0.94	1.07	1.10

Agilent 1100	Elite	2.66	1.65	0.98	1.52	1.04	1.12	0.95	0.95	1.03	1.10
	Agilent	2.83	1.65	1.04	1.51	1.14	1.22	0.94	0.95	1.04	1.04
	Wel Materials	2.65	1.73	0.95	1.47	1.14	1.13	0.91	0.93	1.05	1.02

Agilent 1260	Elite	2.74	1.75	1.06	1.52	1.19	1.26	0.98	0.94	1.14	1.15
	Wel Materials	2.74	1.74	1.10	1.58	1.12	1.23	0.85	0.91	1.03	1.09

Mean		2.67	1.76	1.04	1.49	1.14	1.19	0.93	0.94	1.08	1.10

RSD %		4.25	4.82	4.58	4.22	4.39	4.49	4.90	2.39	4.50	4.74

**Table 6:** RCFs by different columns temperature (*n* = 3).

Temperature/°C	*f*_gallic acid_/*f*_loganin_	*f*_5-HMF_/*f*_loganin_	*f*_morroniside_/ *f*_loganin_	*f*_cornin_/ *f*_loganin_	*f*_sweroside_/ *f*_loganin_	*f*_7α-O-methylmorroniside_/ *f*_loganin_	*f*_7β-O-methylmorroniside_/ *f*_loganin_	*f*_7α-O-ethylmorroniside_/*f*_loganin_	*f*_7β-O-ethylmorroniside_/*f*_loganin_	*f*_cornuside_/*f*_loganin_
25	2.69	1.88	0.98	1.26	1.08	1.04	0.90	0.92	1.15	1.20
30	2.66	1.92	1.00	1.32	1.07	1.08	0.93	0.96	1.16	1.24
35	2.66	1.85	0.98	—	—	1.05	0.96	0.98	1.18	1.23
Mean	2.67	1.88	0.99	1.29	1.08	1.06	0.93	0.95	1.16	1.22
RSD %	0.63	2.01	0.71	3.55	0.79	2.32	3.53	3.24	1.51	1.68

**Table 7:** RCFs by different flow rate (*n* = 3).

Flow rate/mL·min^−1^	*f*_gallic acid_/*f*_loganin_	*f*_5-HMF_/*f*_loganin_	*f*_morroniside_/*f*_loganin_	*f*_cornin_/*f*_loganin_	*f*_sweroside_/*f*_loganin_	*f*_7α-O-methylmorroniside_/*f*_loganin_	*f*_7β-O-methylmorroniside_/*f*_loganin_	*f*_7α-O-ethylmorroniside_/*f*_loganin_	*f*_7β-O-ethylmorroniside_/*f*_loganin_	*f*_cornuside_/*f*_loganin_
0.9	2.61	1.86	0.99	1.26	1.07	1.08	0.93	0.96	1.09	1.22
1.0	2.66	1.92	1.00	1.32	1.07	1.08	0.93	0.96	1.16	1.24
1.1	2.56	1.89	0.99	1.34	1.09	1.09	0.93	0.99	1.20	1.24
Mean	2.61	1.89	0.99	1.31	1.08	1.08	0.93	0.97	1.15	1.23
RSD %	1.90	1.78	0.41	3.29	1.25	0.59	0.19	2.02	4.69	0.77

**Table 8:** Relative retention time of target peaks by different instruments and columns (*n* = 3).

Instruments	Chromatograph columns	*t*_gallic acid_/*t*_loganin_	*t*_5-HMF_/*t*_loganin_	*t*_morroniside_/*t*_loganin_	*t*_cornin_/*t*_loganin_	*t*_sweroside_/*t*_loganin_	*t*_7α-O-methylmorroniside_/*t*_loganin_	*t*_7β-O-methylmorroniside_/*t*_loganin_	*t*_7α-O-ethylmorroniside_/*t*_loganin_	*t*_7β-O-ethylmorroniside_/ *t*_loganin_	*t*_cornuside_/ *t*_loganin_
Waters 2695-2998	Elite	0.16	0.21	0.51	0.95	0.97	1.19	1.20	1.46	1.47	1.59
	Agilent	0.16	0.22	0.51	0.94	0.96	1.23	1.25	1.52	1.54	1.67
	Wel Materials	0.16	0.22	0.57	0.97	0.98	1.17	1.19	1.43	1.46	1.58

Agilent 1100	Elite	0.16	0.21	0.52	0.96	0.98	1.18	1.19	1.45	1.46	1.59
	Agilent	0.17	0.20	0.51	0.95	0.97	1.20	1.21	1.47	1.50	1.63
	Wel Materials	0.16	0.24	0.57	0.97	0.99	1.16	1.19	1.43	1.45	1.58

Agilent 1260	Elite	0.16	0.22	0.53	0.96	0.98	1.18	1.20	1.46	1.47	1.60
	Wel Materials	0.17	0.21	0.56	0.97	0.99	1.17	1.19	1.44	1.46	1.58

Mean		0.16	0.22	0.53	0.96	0.98	1.19	1.20	1.46	1.48	1.60

RSD %		3.28	4.48	4.90	1.02	0.85	1.87	1.72	2.06	2.00	1.95

**(a):** 

Number	Loganin	Gallic acid		5-HMF		Morroniside		Cornin		Sweroside	
	External standard	External standard	QAMS	External standard	QAMS	External standard	QAMS	External standard	QAMS	External standard	QAMS
1	7.32	0.50	0.54	—	—	8.22	8.25	0.26	0.27	0.87	0.90
2	7.45	0.39	0.41	—	—	5.53	5.52	0.25	0.27	0.90	0.92
3	7.30	0.53	0.58	—	—	6.28	6.29	0.26	0.28	0.87	0.90
4	6.94	0.45	0.49	—	—	5.35	5.34	0.19	0.19	0.66	0.67
5	7.11	0.67	0.73	—	—	4.85	4.84	0.20	0.21	0.73	0.75
6	7.78	0.45	0.48	—	—	6.49	6.50	0.22	0.23	0.78	0.80
7	7.54	0.43	0.46	—	—	10.65	10.70	0.25	0.26	0.83	0.85
8	7.38	0.41	0.44	—	—	6.37	6.37	0.27	0.28	0.74	0.76
9	6.86	0.42	0.45	—	—	8.85	8.88	0.24	0.25	0.87	0.90
10	7.60	0.40	0.43	—	—	7.85	7.87	0.26	0.27	0.92	0.95
11	6.22	0.50	0.54	—	—	7.88	7.91	0.27	0.28	0.86	0.88
12	7.64	0.54	0.59	—	—	4.79	4.78	0.20	0.21	0.80	0.83
13	7.13	0.45	0.49	—	—	3.50	3.48	0.18	0.19	0.72	0.74
14	6.89	1.15	1.28	—	—	7.30	7.32	0.28	0.30	0.93	0.96
15	6.47	1.10	1.23	—	—	7.77	7.79	0.27	0.28	0.86	0.88
16	6.87	0.96	1.07	—	—	10.41	10.46	0.28	0.30	0.89	0.92
17	6.28	1.07	1.20	—	—	7.49	7.51	0.28	0.29	0.86	0.89
18	6.26	1.06	1.19	—	—	7.89	7.91	0.27	0.28	0.86	0.89
19	6.58	1.39	1.56	—	—	6.96	6.97	0.27	0.29	0.97	1.00
20	6.26	1.13	1.26	—	—	6.52	6.53	0.27	0.29	0.86	0.88
21	7.01	1.27	1.42	—	—	8.04	8.07	0.29	0.30	0.91	0.94
22	6.63	2.15	2.43	1.67	1.71	9.41	9.45	0.29	0.30	0.95	0.98
23	6.49	2.10	2.38	2.07	2.12	10.21	10.26	0.28	0.30	0.96	0.99
24	7.92	2.40	2.72	1.42	1.45	13.52	13.59	0.34	0.36	1.10	1.14
25	6.86	1.28	1.44	1.81	1.86	8.76	8.79	0.22	0.23	0.76	0.78
26	7.95	1.30	1.46	2.04	2.10	11.32	11.37	0.25	0.26	0.87	0.90
27	6.28	1.97	2.23	1.91	1.96	9.57	9.62	0.28	0.30	0.85	0.87
28	6.60	2.01	2.27	1.94	1.99	10.88	10.94	0.30	0.31	0.91	0.94
29	6.44	2.11	2.39	1.66	1.70	10.97	11.03	0.27	0.29	0.81	0.83
30	6.67	2.01	2.27	1.71	1.75	11.45	11.51	0.29	0.31	0.77	0.79
31	8.63	1.34	1.50	2.43	2.49	14.02	14.09	0.31	0.32	0.89	0.92

**(b):** 

Number	7*α*-O-Methylmorroniside		7*β*-O-Methylmorroniside		7*α*-O-Ethylmorroniside		7*β*-O-Ethylmorroniside		Cornuside	
	External standard	QAMS	External standard	QAMS	External standard	QAMS	External standard	QAMS	External standard	QAMS
1	0.02	0.02	0.07	0.07	—	—	—	—	1.30	1.32
2	0.01	0.01	0.05	0.04	—	—	—	—	1.28	1.31
3	0.01	0.01	0.08	0.08	—	—	—	—	1.26	1.28
4	0.01	0.01	0.05	0.04	—	—	—	—	1.17	1.19
5	0.03	0.03	0.08	0.07	—	—	—	—	1.23	1.25
6	0.02	0.01	0.06	0.05	—	—	—	—	1.65	1.68
7	0.02	0.01	0.07	0.07	—	—	—	—	1.65	1.68
8	0.01	0.01	0.07	0.06	—	—	—	—	1.66	1.69
9	0.01	0.01	0.05	0.05	—	—	—	—	1.55	1.58
10	0.01	0.01	0.04	0.04	—	—	—	—	1.74	1.77
11	0.01	0.01	0.06	0.06	—	—	—	—	0.96	0.97
12	0.01	0.01	0.05	0.04	—	—	—	—	1.20	1.22
13	0.01	0.01	0.04	0.03	—	—	—	—	1.01	1.03
14	0.01	0.01	0.07	0.06	—	—	—	—	1.04	1.05
15	0.01	0.01	0.06	0.06	—	—	—	—	1.00	1.01
16	0.01	0.01	0.07	0.06	—	—	—	—	1.10	1.12
17	0.02	0.02	0.06	0.06	—	—	—	—	0.98	1.00
18	0.01	0.01	0.06	0.06	—	—	—	—	0.95	0.96
19	0.01	0.01	0.07	0.06	—	—	—	—	0.98	0.99
20	0.02	0.02	0.06	0.05	—	—	—	—	0.95	0.96
21	0.02	0.02	0.07	0.07	—	—	—	—	1.03	1.04
22	0.01	0.01	0.07	0.07	0.14	0.14	0.23	0.23	0.81	0.82
23	0.02	0.02	0.07	0.06	0.15	0.15	0.25	0.25	0.86	0.87
24	0.02	0.02	0.09	0.08	0.20	0.19	0.30	0.30	1.10	1.12
25	0.01	0.01	0.05	0.05	0.24	0.24	0.28	0.28	1.11	1.12
26	0.01	0.01	0.05	0.04	0.24	0.24	0.27	0.27	1.27	1.28
27	0.03	0.03	0.09	0.08	0.24	0.24	0.27	0.27	0.87	0.88
28	0.01	0.01	0.06	0.06	0.24	0.23	0.28	0.28	0.94	0.95
29	0.02	0.02	0.08	0.07	0.23	0.22	0.24	0.24	0.94	0.95
30	0.02	0.02	0.07	0.07	0.20	0.19	0.23	0.22	0.96	0.97
31	0.02	0.02	0.09	0.09	0.35	0.34	0.38	0.38	1.75	1.78
